# Evaluating integrated headache care: a one-year follow-up observational study in patients treated at the Essen headache centre

**DOI:** 10.1186/1471-2377-11-124

**Published:** 2011-10-10

**Authors:** Charly Gaul, Julia Brömstrup, Günther Fritsche, Hans C Diener, Zaza Katsarava

**Affiliations:** 1 Headache Centre, Department of Neurology, University Duisburg-Essen, Essen, Germany

## Abstract

**Background:**

Outpatient integrated headache care was established in 2005 at the Essen Headache Centre in Germany. This paper reports outcome data for this approach.

**Methods:**

Patients were seen by a neurologist for headache diagnosis and recommendation for drug treatment. Depending on clinical needs, patients were seen by a psychologist and/or physical therapist. A 5-day headache-specific multidisciplinary treatment programme (MTP) was provided for patients with frequent or chronic migraine, tension type headache (TTH) and medication overuse headache (MOH). Subsequent outpatient treatment was provided by neurologists in private practice.

**Results:**

Follow-up data on headache frequency and burden of disease were prospectively obtained in 841 patients (mean age 41.5 years) after 3, 6 and 12 months. At baseline mean headache frequency was 18.1 (SD = 1.6) days per month, compared to measurement at 1 year follow-up a mean reduction of 5.8 (SD = 11.9) headache days per month was observed in 486 patients (57.8%) after one year (TTH patients mean: -8.5 days per month; migraine mean: -3.2 days per month, patients with migraine and TTH mean: -5.9 days per month). A reduction in headache days ≥ 50% was observed in 306 patients (36.4%) independent of diagnosis, while headache frequency remains unchanged in 20.9% and increase in 21.3% of the patient.

**Conclusion:**

Multidisciplinary outpatient headache centres offer an effective way to establish a three-tier treatment offer for difficult headache patients depending on clinical needs.

## Background

Headache disorders, especially migraine and tension-type headache (TTH), not only result in severe individual burden but also have serious socioeconomic impact [1,2]. High costs for the health care system as well as reduced work capacity and impairments in social life indicate the relevance of these headache disorders [3]. Frequent headaches lead patients to repeated hospitalizations, consultation of different medical specialists and expenditure on alternative therapies due to the fact that their understanding of headache may be based on dysfunctional conceptions and lack of information. Standard care for headache is provided by general practitioners in Germany, only a part of the patients are treated by neurologists. Most of primary care physicians and neurologists are not specialized in headache care. In general, treatment of chronic migraine, TTH and medication overuse headache (MOH) is challenging for general practioners and neurologists in private practice. As a consequence, many patients seek help from different disciplines (dentists, otorhinolaryngologists, orthopedic surgeons, ophthalmologists etc.). Incorrect diagnosis and thus unsuccessful treatment result in high costs. Obviously an unstructured concept of health care is not sufficient for patients with frequent or difficult-to-treat headaches. In fact, the recommended access to behavioural psychologists or physiotherapists is not available within the standard healthcare system. To overcome these difficulties in daily practice, integrated headache care was developed and established by health insurance companies and the University Hospital in Essen, Germany in 2005. Integrated headache care comprises different disciplines (neurologists, behavioural psychologists, physiotherapists) and close collaboration between hospital, outpatient headache center and private practice. This follows the suggestions of a three-tier interdisciplinary system for headache care [4-6]. Integrated headache care started in 2005 in cooperation with selected health insurance companies and was expanded over time [7].

In this report prospectively collected baseline and one-year follow-up data from patients with difficult to treat headaches are reported focussing on changes in headache frequency from baseline to follow-up and predictors of outcome and treatment success.

## Methods

This is a prospective observational study reporting the first experiences and the outcome of the headache treatment programme of the West German Headache Centre established in 2005. Due to the observational and non-interventional character of the evaluation, no randomization to different treatment modalities was done. Inclusion criteria were a) age > 18 years b) diagnosis of migraine, TTH, combination headache and/or MOH according to ICHD-II [8] and c) adequate knowledge of the German language. The project was approved by the local Ethics Committee. Informed consent was obtained from all patients. All patients were seen at the outpatient headache clinic by experienced board certified neurologists who diagnosed the headache type according to ICHD-II [8] and collected information about demography and frequency and intake of acute and preventive drugs.

### Setting and Concept of the West German Headache Centre

The West German Headache Centre provides an outpatient and day clinic service for patients with frequent and/or difficult-to-treat headaches who are referred by neurologists, general practioners or their insurance company. The multidisciplinary team of the Headache Centre consists of neurologists, physical and sports therapists, behavioural psychologists, headache nurses as well as consultants from other disciplines if needed [5]. Patients suffering from episodic headache with low headache frequency were given a treatment recommendation for subsequent therapy by their general practioner or neurologist in private practice. Patients suffering from more frequent or chronic headache, high frequency intake of medicine for the treatment of acute headache episodes (analgesics, NSAID, ergotamine, triptans), medication overuse headache (MOH) or whose current medication was insufficient were selcted for more intensive therapy. In a first step, they consulted a psychologist and/or a physical therapist. The decisions about these referrals were taken by the neurologist based on disease history and clinical findings. Initial sessions with psychologists consist of a one-hour face-to-face contact. For some patients this resulted in treatment recommendations. Most patients received education about stress, specific lifestyle changes and other self-help options. Referral to the physiotherapists consists mainly in evaluation of the musculo-skelettal system and education about aerobic endurance training as a preventive treatment for headache. Difficult-to-treat patients are referred to the outpatient multidisciplinary day clinic treatment programme (MTP). The MTP's multidisciplinary team consists of neurologists, physical and sports therapists as well as psychologists. Patients are educated in how to handle headache in everyday life by learning strategies of stress management, muscle relaxation and sports. The 5-day programme is scheduled in different sessions that focus on education about headache (symptoms, etiology and pathophysiology), on treatment options and on the correct use of headache medication. Details of the treatment programme were published elsewhere [9].

### Baseline and outcome data

Baseline data were obtained in a structured face-to-face interview and recorded in a custom-made data base. Follow-up was performed by three telephone interviews (after 3, 6 and 12 months) performed by trained medical students. Demographic and personal data (age, sex, weight, martial-status, education level) were obtained as well as the prior and present use of attack-aborting and prophylactic medication (data not shown). The questionnaire comprised data on headache frequency, and burden of disease measured by MIDAS (Migraine disability Assesment Score) questionnaire [10]. Data were analyzed for predictors of treatment success (defined as ≥ 50% headache reduction per month).

### Statistical analysis

Binary logistic regression was employed to determine predictors influencing primary outcome, which was defined as a reduction of > 50% in headache days per month, resulting in odds ratios (OR) with 95% confidence intervals (CI). The influence of the following variables: age (< 40 vs. ≥ 40 years; younger half vs. older half of the patients), smoking status (smoker vs. non-smoker), weight (BMI < 25 vs. ≥ 25), education level (low education vs. high education), headache diagnosis (migraine vs TTH vs combination of migraine and TTH) treatment in the MTP, headache frequency at baseline (0-5 days, 6-10 days, 11-15 days, 16-20 days, 21-25 days, 26-30 days) and family status (single vs. partnership) were calculated. Chi-square tests were used for comparison of categorical variables. P-value below 0.05 was defined as significant. PASW Statistics 18.0.0 was used for statistics analysis.

## Results

### Patient characteristics

841 of 1871 patients visiting the headache centre were interviewed at 3, 6 and 12 months. The others refused the interviews, provided insufficient data or were lost to follow-up. We include those patients in which baseline demographics (age, gender), data on diagnosis, and headache days (admission and follow-up) were available. Mean age was slightly different between included vs the non-included patients (40.9 vs 39.5 years). The mayority of patients were female within the non-included (85.7%) and the presented patients (81.9%). Therefore a representative subgroup not clinically different from the total population treated in the headache centre is presented.

**Figure 1:**
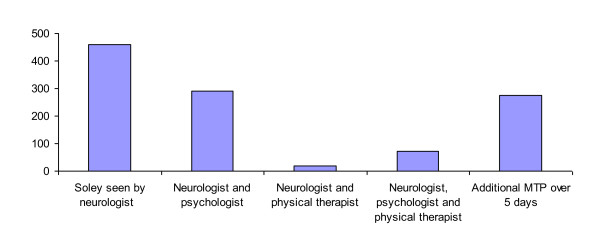
**Allocation to different cobinations of consultation and additional MTP**. MTP = Multidisciplinary treatment

All patients were seen by a neurologist. In addition, 362 (43.0%) were seen by a psychologist, 94 (11.2%) consultated a physical therapist, and 274 patients (32.6%) participated the MTP. Allocation to different cobinations of consultation and additional MTP was displayd (Figure 1). The demographic and diagnostic characteristics of the 841 patients included are displayed in table 1. The burden of disease was measured by MIDAS questionnaire. Seventy-five percent of patients suffered from migraine, 15.7% were diagnosed with migraine and TTH and 8.5% were suffering from TTH. The highest headache frequency at admission was reported for patients with TTH (25.6 headache days per month, mean). Patients with migraine and TTH had an average of 16.7 headache days per month and patients suffering from migraine had a mean headache frequency of 8.9 days per month. The mean duration of disease was 11.7 years.

**Table 1:** Demographic and Clinical Characteristiscs

		Migraine (n = 637)	Migraine + TTH (n = 132)	TTH (n = 72)
Gender n (%)	Male	85 (13.3)	11 (8.3)	23 (31.9)
	
	Female	552 (86.7)	121 (91.7)	49 (68.1)

Age, mean (SD)		40.7 (12.7)	38.6 (13.7)	45.3 (17.8)

Education* n (%)	Low education	350 (54.9)	69 (52.3)	29 (40.3)
	
	High education	193 (30.3)	44 (33.3)	10 (13.9)
	
	Missíng	94 (14.8)	19 (14.4.)	33 (45.8)

Employment, n (%)	Unemployed	30 (4.7)	5 (3.8)	8 (11.1)
	
	Fulltime	441 (69.2)	90 (68.2)	35 (48.6)
	
	Housemaker	68 (10.7)	13 (9.8)	7 (9.7)
	
	Pensioner	33 (5.2)	10 (7.6)	17 (23.6)
	
	Missing	65 (10.2)	14 (10.6)	5 (6.9)

Marital Status, n (%)	Single	81 (12.7)	22 (16.7)	12 (16.7)
	
	Partnership	421 (66.1)	77 (58.3)	44 (61.1)
	
	Others	42 (6.6)	12 (9.1)	7 (9.7)
	
	Missing	93 (14.6)	21 (15.9)	9 (12.5)

Smoking, n (%)	Yes	91 (14.3)	16 (12.1)	11 (15.3)
	
	No	448 (70.3)	96 (72.7)	47 (65.3)
	
	Missing	98 (15.4)	20 (15.2)	14 (19.4)

Duration of disease, years mean (SD)		19.9 (12.5)	17.2 (13.5)	11.7 (11.5)

Headache frequency at admission days/month, mean (SD)		8.9 (19.9)	16.7 (31.8)	25.6 (34.0)

MIDAS grade** at	I	20.2 (20.6)	26.3 (26.4)	n.a.
	
admission, mean (SD)	II	18.2 (17.2)	21.7 (21.0)	n.a.
	
	III	20.6 (16.4)	22.0 (18.7)	n.a.
	
	IV	36.1 (22.9)	37.9 (24.2)	n.a

TTH: Tension Type Headache; n.a.: not applicable. * Education level is divided into two groups: a) Low education: no school qualification+ high school qualification. b) High education: A-level+ University degree, ** MIDAS: Migraine Disability Assessment Score. MIDAS scores are categorized by disability levels: little or no disability (grade I: 0-5), mild disability (grade II: 6-10), moderate disability (grade III: 11-20), severe disability (grade IV: 20+); S.D.: standard deviation, n: number of patients.

### Outcome and predictors

**Figure 2:**
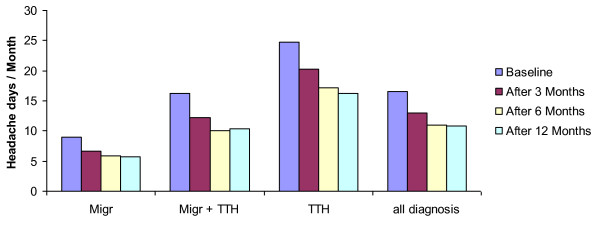
**Course of headache days during one year**. Reduction of headache days per month during one year, grouped for diagnosis and different dates of exploration. (Migr = migraine, TTH = tension-type headache)

An overall reduction of headache days per month was observed in 486 patients (57.8%) after one year (Figure 2). Mean reduction in headache frequency was 5.8 (SD = 11.9) days per month compared to baseline headache frequency. Unchanged frequency was reported in 176 patients (20.9%), while an increase of headache days after one year was reported by 179 patients (21.3%).

The primary outcome (reduction in headache days ≥ 50% after one year) was observed in 306/841 patients (36.4%). The analysis identified two main factors for favourable outcome: headache frequency at baseline and age. No other factors (gender, education, smoking, BMI) (Table 2) were statistically significant.

**Table 2:** Predictive factors for favourable outcome (> 50% reduction of headache days/month) of the treatment programme

Risk Factors (groups, number of patients)		Univariate Odds Ratio (95% CI) P-value	Multivariate Odds Ratio (95% CI) P-value
Age (< 40 years, 367; > 40 years; 471)		**Referent** **1.8** **(1.35 - 2.39) < .001**	**Refererent** **1.93** **(1.33 - 2.79) < .001**

Nicotin (yes, 118; no, 588)		0.71 (0.48 - 1.07) 0.10	0.75 (0.48 - 1.18) 0.21

BMI (> 25, 288; < 25, 543)		0.92 (0.69 - 1.24) 0.58	0.98 (0.67-1.43) 0.90

Education (low education, 510; high education, 246)		0.97 (0.71 - 1.33) 0.85	1.02 (0.70-1.50) 0.90

MTP (no, 557; yes, 273)		**0.73 (0.54 - 0.97) 0.05**	0.70 (0.47-1.02) 0.06

Headache frequency at baseline (number of patients)	0-5 days (251)	1.0 Referent	1.0 Referent
	
	6-10 days (255)	**1.39 (0.94 - 2.04) < .001**	**1.56 (0.99-2.47) 0.05**
	
	11-15 days (145)	**1.89 (1.22 - 2.93) .004**	**2.08 (1.24-3.51) < .001**
	
	16-20 days (49)	**5.03 (2.64 - 9.61) < .001**	**7.42 (3.29-16.75) < .001**
	
	21-25 days (30)	**4.38 (2.00 -9.60) < .001**	**8.65 (3.14-23.84) < .001**
	
	26-30 days (108)	**2.92 (1.82 - 4.69) < .001**	**6.57 (3.20-13.49) < .001**

Headache diagnosis (n)	Migraine (635)	1.0 Referent	1.0 Referent
	
	Migraine + TTH (131)	1.14 (0.77 - 1.68) 0.51	0.69 (0.41-1.17) 0.17
	
	TTH (72)	1.07 (0.65 - 1.77) 0.79	0.34 (0.16-0.73) 0.005*

Family (single, 175; partnership, 540)		**0.69 (0.49 - 0.98) 0.04**	0.78 (0.52-1.17) 0.23

Patients with migraine, TTH and migraine and TTH are included. * Only measurements which show significance in the univariate analysis and could be confirmed in the multivariate analysis were were accepted as outcome predictors

There was no significant difference regarding the primary outcome between the headache groups. However, regarding the absolute reduction of headache days, TTH patients improved most (mean: -8.5 days per month) compared to patients with migraine (mean:-3.2 days per month) and patients with migraine and TTH (mean: -5.9 days per month).

The course of headache frequency during the one-year follow-up was analyzed regarding worsening from episodic (< 15 headache days/month) to chronic (> 15 headache days/month) as well as recovery from chronic to episodic headache. Five-hundred sixty-three (67%) patients remained episodic, while 25 patients showed a progression to chronic migraine. In 177 (21%) patients, the headache frequency declined from a chronic to an episodic course. Persistence of chronic headache during one year was documented in 76 patients (9%).

### Predictors for primary outcome

Table 2 summarizes the univariate as well as the multivariate predictive factors for favourable outcome (≥ 50% reduction of headache days/month).

### Course of disease in patients suffering from medication overuse headache

Fifty-nine of 841 patients were documented with MOH in one of the questionnaires from baseline to follow-up 3. Fifty patients were documented with MOH only at baseline. Fivty-three of 59 patients (91.4%) who were diagnosed with MOH were in the low-education group. Six (10%) patients showed persistence of MOH during one year (from baseline to follow-up 3). At the one-year follow up, three patients (5%) were identified with a newly-developed MOH.

### Burden of disease

**Figure 3:**
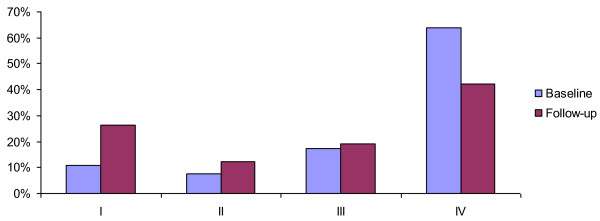
**Burden of disease measured with MIDAS**. MIDAS (Grade I-IV) of all patients, comparison between baseline and 1-year follow-up

Regarding burden of disease, 64% of the patients presented with MIDAS IV at admission, decreasing to 42.2% after 12 months. As a consequence, MIDAS I increased from 11% to 26.3% after 12 months (Figure 3).

## Discussion

Integrated headache was established to provide multidisciplinary treatment to improve treatment for difficult-to-treat headache patients. Prospectively collected observational data of a large cohort of headache patients treated in our tertiary headache center showed overall improvement of headache frequency in 57.8% of the patients. Thirty-six percent of patients achieved a ≥ 50% reduction of headache days per month irrespective of primary headache subgroup. In contrast no improvement (20.9%) or worsening (21.3%) was reported in remaining patients. Headaches usually show a fluctuating headache frequency influenced by different factors (for example psychiatric comorbidity, stress, live-events) it is expectable that a part of them did not improve or wosen during time course. As shown in a recent study of our group adherence to live style modifications predict primary outcome [9]. It is not surprising that short or single interventions in a headache center did not result in improvement of headaches in all patients. In absolute terms reduction of headache days in TTH was more pronounced than in migraine. Multivariate analysis revealed best outcome for patients older than 40 years compared to the younger. We could not explain this finding. Another important variable is the number of headache days per month at baseline. Patients with migraine and migraine and TTH who suffered from nearly daily headache (> 25 headache days per month) showed the best outcome, whereas patients with the lowest number of headache days (0-5) at baseline showed almost no change in headache frequency.

Regarding offerd consulation by neurologist and if possible once in addition by a physical therapists and/or a psychologist differentiation on outcome one year later can not be expected. Data does not suggest that additional MTP resulted in a better outcome. However, the study was not designed to compare these strategies. MTP treatment was indicated by headache frequency and burden of disease, but it was only available if treatment costs were covered by the health insurance. In addition treatment in the MTP depends on motivation of the patient and living distance to the headache center. Therefore treatment allocation was not only determined by medical aspects. Independet from this we recommend MTP especially in patients suffering from MOH. Succesful treatment of MOH resulting in reduction of the reported hiph relapse rate was shown in recent published data due to MTP [9]. This might be explained by specific education and main emphases on MOH during the MTP.

Jensen et al. performed a 2-year systematic follow-up in the Danish Headache Centre in order to characterize patients and treatment results. They identified predictors for good outcome as female gender, migraine as primary headache, triptan-overuse and a mean headache frequency of 10 days/month, whereas tension-type headache and overuse of simple analgesics predicted poor outcome [11]. These results are in some aspects contradictory to our findings. We found no significant influence of gender or headache type on primary outcome (≥ 50% reduction of headache frequency). In a population-based longitudinal study Bigal et al. assessed the influence of baseline body mass index on the response to headache preventive treatment. Patients suffering from episodic, chronic or transformed migraine who sought care in a headache clinic were included. Baseline information included headache frequency, number of days with severe headache and headache-related disability. The same information was obtained after three months of preventive treatment. After treatment, headache frequency declined in the entire population but no significant differences were found among BMI groups. Furthermore, BMI did not account for changes in disability, headache frequency, or in the number of days with severe headache per month [12]. These results are in line with our data in showing no significant difference in BMI groups and reduction of headache days with categorization of BMI into only two subgroups (< 25 vs. ≥ 25) and a longer follow-up (twelve months). High BMI was a risk factor for increasing headache frequency in some studies [13,14] but not others [15,16]. Moreover, we could not find an association between smoking and reduction of headache days. These findings are supported by a metanalysis of three studies focusing on the association of lifestyle factors (BMI, alcohol, smoking and physical activity) and headache prevalence in Germany which also found no association between migraine and obesity or smoking [17].

In order to investigate the association between level of education and reduction of headache frequency, the education level was split into a high and low education group. There was no significant difference between level of education and reduction of headache frequency (≥ 50% reduction). However, 91.4% who were diagnosed with MOH were in the low-education group, indicating that a lower level of education may be a risk factor for overuse of medication. This is supported by observations from Atasoy et al. who evaluated headache characteristics, socioeconomic and educational variabilities in subgroups of MOH and migraine patients. Their data showed that the frequency of migraine attacks as well as the duration of MOH and lower income was more frequent in low-educated migraine-patients [18]. Scher et al. identified level of education as factor of headache chronification [13], which has been shown for MOH, too [19]. Furthermore, we did not find an association of marital status and reduction of headache days. Being married was associated with better prognosis in another study [13]. The consequence of these observations is that no patients should be excluded from integrated headache care based on baseline variables.

We showed cost effectiveness for integrated headache care treatment [20]. A formal socioeconomic analysis was not part of the present study and will be the focus of future research.

Strength of our study is the prospective design, the large cohort and the long follow-up period (12 months). It is important to investigate long-term effects of headache therapy beyond the 3 months observation time in clinical trials. Changes in medical treatment as well as changes in lifestyle and behaviour need time to show an impact on headache frequency. One of the major limitations of the study is the significant number of patients lost to follow-up and the high rate of dropouts, as well as the fact that we could not present a parallel group design with a cohort receiving standard care from general practitioners. Moreover this is a prospective observational study and not a controlled trial, which has impact on completeness of the data therefore missings were indicated in the tables. There is a clear need for future studies randomizing patients to different treatment modalities to prove the best care. Moreover, the study population was biased and taken from a tertiary headache centre taking care of severely affected and more chronic headache patients. Thus, study results may not be easily generalized to the headache population.

## Conclusion

In summary, a large number of headache patients and their response to treatment within a newly-developed integrated headache care concept in Germany were studied. Multidisciplinary treatment resulted in a reduction of headache frequency (≥ 50%) especially in older patients (> 40 years) and in patients with a high number of headache days at baseline. Regarding burden of disease, it has been demonstrated that greatly-affected patients (MIDAS IV) improved during one year. Interdisciplinary care has been successfully established and it is probably even cost-effective. Integrated care is an efficient way to take care of the individual needs of each severly affected headache patient.

## Competing interests

The authors declare that they have no competing interests.

## Authors' contributions

All authors made a substantial contribution to this work. CG, HCD, GF and ZK conceived and designed the study. CG, JB and GF conducted the analysis and drafted the manuscript. HCD revised the manuscript carefully. All authors participated in the interpretation of data, revised the manuscript and approved the final version of the manuscript that is now being submitted for publication.

## Pre-publication history

The pre-publication history for this paper can be accessed here:

http://www.biomedcentral.com/1471-2377/11/124/prepub
